# Predicting gastrointestinal and renal involvement in adult IgA vasculitis

**DOI:** 10.1186/s13075-019-2089-2

**Published:** 2019-12-26

**Authors:** Alojzija Hočevar, Matija Tomšič, Vesna Jurčić, Katja Perdan Pirkmajer, Žiga Rotar

**Affiliations:** 1 Department of Rheumatology, University Medical Centre Ljubljana, Vodnikova cesta 62, 1000 Ljubljana, Slovenia; 2 Faculty of Medicine, University of Ljubljana, Ljubljana, Slovenia; 3 Faculty of Medicine, Institute of Pathology, University of Ljubljana, Ljubljana, Slovenia

**Keywords:** IgA vasculitis, Gastrointestinal involvement, Renal involvement

## Abstract

**Background:**

Immunoglobulin A vasculitis (IgAV) is still poorly defined in the adult population. We aimed to determine the predictors of gastrointestinal (GI) or renal involvement in adult IgAV.

**Methods:**

The prospective study included histologically proven adult IgAV cases diagnosed between January 2013 and July 2019 at our secondary/tertiary rheumatology center. We evaluated the role of clinical and the laboratory parameters as markers predicting the GI or renal involvement in IgAV, using the multiple logistic regression analysis.

**Results:**

During the 79-month observation period, we identified 214 new adult IgAV cases (59.3% males, median (interquartile range) age 64.6 (57.2–76.7) years). The GI tract and renal involvement developed in 58 (27.1%) and 83 (38.8%) cases, respectively (concurrently in 26 (12.1%) cases). In the multivariate logistic regression analysis, generalized purpura (OR 6.74 (95%CI 3.18–14.31)), the pre-treatment neutrophil to lymphocyte ratio (NLR) > 3.5 (OR 2.78 (95%CI 1.34–5.75)), and elevated serum IgA levels (OR 0.40 (95%CI 0.20–0.79)) were extracted as factors associated with GI complications, whereas current smoking (OR 3.23 (95%CI 1.50–6.98)), generalized purpura (OR 1.98 (95%CI 1.08–3.61)), elevated serum IgA (OR 2.25 (95%CI 1.21–4.18)), NLR > 3.5 (OR 1.96 (95%CI 1.02–3.77)), and marginally age (1.02 (95%CI 1.01–1.04)) emerged as factors associated with renal complications.

**Conclusion:**

Generalized purpura and pre-treatment NLR predicted both GI and renal involvement, whereas active smoking was associated with renal involvement, and the serum IgA level had a divergent effect on renal and GI involvement in adult IgAV.

## Introduction

Immunoglobulin A vasculitis (IgA) is an immune complex small vessel leukocytoclastic vasculitis that commonly affects the skin, joints, gastrointestinal (GI) tract, and kidneys [1]. IgAV is a typical childhood vasculitis, commonly with a benign, self-limiting course and complete recovery in the young population. In adults, a severe acute disease with significant GI and renal involvement is common [2, 3]. GI involvement with a severe hemorrhage or bowel perforation represents the major risk of mortality in acute adult IgAV, and renal involvement is associated with an increased risk of progression to chronic kidney failure [2]. Nevertheless, the predictors of visceral involvement in acute adult IgAV have not been extensively studied. Cao et al. reported that the risk of significant renal disease increased with patient age, and the presence of necrotic or bullous skin lesions [4]. Patient age forecasted visceral involvement also in the study by Poterucha et al. Adults under the age of 40 had an increased risk of GI involvement, and those over 40 without eosinophils in a skin biopsy had an increased risk of renal involvement [5]. Contrary to St John et al., who reported a predictive value of skin lesions on the upper and lower extremities for significant long-term renal involvement [6], the distribution of skin lesions above the waist was not found to be a reliable indicator of systemic disease in adult IgAV in the study by Poterucha et al. [5]. Nagy et al. described in a retrospective study a significant association between pre-treatment elevated neutrophil to lymphocyte ratio (NLR) and the development of GI or renal manifestations [7]. A similar association between an elevated NLR and severe GI or nephritis has recently been described also in childhood IgAV [8].

The aim of our study was to determine the predictors of gastrointestinal and renal involvement in a prospectively collected cohort of adult IgAV.

## Methods

### Setting and patient selection

This prospective study was conducted at the Department of Rheumatology, University Medical Centre Ljubljana, Slovenia. Patients with suspected vasculitis are regularly referred by their general practitioners or other subspecialists to our early intervention clinic, where they are evaluated on the day of the referral, obviating the need for immunosuppressive treatment before referral.

We included adults (i.e., persons aged ≥ 18 years), with the symptoms and signs compatible with the definition of IgAV according to the 2012 revised International Chapel Hill Consensus Conference Nomenclature of Vasculitides [1], with histologically proven IgAV, diagnosed for the first time between January 2013 and August 2019. Additionally, all patients fulfilled the classification criteria of the European League Against Rheumatism/Paediatric Rheumatology International Trials Organization/Paediatric Rheumatology European Society (EULAR/PRINTO/PRES) criteria for IgAV [9].

### Clinical, laboratory, and histological data

Patients underwent a detailed clinical evaluation (including a structured history of smoking and potential triggers of IgAV, i.e., infection or the use of new medication within a month prior to an IgAV episode, and cancer history), and an extensive laboratory workup, as described previously [10]. The definitions used for the assessment of the skin, renal, and gastrointestinal involvement are provided in Table 1.

**Table 1:** Definitions of skin, kidney, and gastrointestinal involvement in IgAV

Purpura	
Localized	Vasculitic lesions present only below the waistline
Generalized	Vasculitic lesions extending above the waistline
Renal involvement	
Hematuria	> 5 red blood cells per high power field or red blood cells casts in the urinary sediment or hemoglobinuria ≥ 2+ on dipstick
Macrohematuria	> 1500 red blood cells/mm^3^ of urine
Proteinuria	urine protein excretion > 300 mg/day
Severe involvement	nephrotic or nephritic syndrome with an acute worsening of the renal function, defined as either an increase in serum creatinine concentration or a decrease in the glomerular filtration rate estimated by the MDRD-4 > 25% from the patient’s baseline
GI involvement	new onset of diffuse abdominal pain or gastrointestinal bleeding
Severe involvement	bloody diarrhea, ileus or bowel perforation

*GI* gastrointestinal, *MDRD-4* Modification of Diet in Renal Disease, 4 variables

The serum immunoglobulin A (IgA) concentration, pre-treatment white blood cell count (WBC), and NLR were measured. Since concurrent infection could influence the NLR, concurrent infections were recorded and included in the analysis.

Skin or renal biopsies were evaluated using bright field microscopy, and direct immunofluorescence.

### Statistical analysis

The results were expressed as a median and interquartile range (IQR) or mean and standard deviation (SD) for metric, and as percentages for categorical variables. To test the differences between IgAV subgroups, we used the Mann-Whitney test for metric and Fisher’s exact test for categorical variables. A receiver operating characteristic (ROC) curve was constructed to evaluate the prognostic utility of NLR and to determine the optimal cut-off value. Potential predictors of IgAV GI and renal involvement with a *p* value < 0.2 were tested using a multiple logistic regression analysis. The significance threshold selected for the final model was set at 0.05.

## Results

### Demographic, epidemiological data, and classification criteria

During the 79-month observation period, we identified 214 new IgAV cases (127 (59.3%) males). The median (IQR) patient age at diagnosis was 64.6 (47.2–76.7, range 18–96) years. There were 42 (19.6%) current smokers (daily smoking on average (SD) 14 (9) cigarettes) and 55 (25.7%) past smokers. Sixty-nine (32.2%) and 52 (24.3%) patients reported prior infection or the use of new medication within a month of the IgAV episode onset, respectively. In 33 (15.4%) patients, a concurrent infection was recorded. Twenty-seven (12.6%) patients had a history of cancer. The median (IQR) symptom duration before diagnosis was 7 (5–21) days. One-hundred-and-eighty-five (86.4%) patients presented with purpura for the first time, while 29 recalled previous episodes of similar skin lesions without a definitive diagnosis in the past.

All 214 patients fulfilled EULAR/PRINTO/PRES classification criteria for IgAV [9]. In addition to skin involvement, in all 214 patients, the skin biopsy was performed and was consistent with IgAV. Sixty-seven out of 214 (31.3%) patients had skin limited IgAV (i.e., fulfilling mandatory skin criterion and histological criterion); the remaining 68.7% fulfilled at least one additional item of EULAR/PRINTO/PRES classification criteria for IgAV (i.e., articular or GI or renal item).

### IgAV features

Skin involvement with histologically proven IgAV was present in all 214 patients. Necrotic and bullous lesions developed in 98 (45.8%) patients. In 105 (49.1%) patients, purpura was limited to the lower limbs or up to waist level. Vasculitic lesions above the waistline (i.e., generalized purpura) were observed in 109 (50.9%) patients. Seventy-two (33.6%) patients reported arthralgia and 29 (13.6%) developed arthritis. We recorded GI involvement in 58 (27.1%) patients, and 26 (12.1%) of them had concurrent renal involvement. Forty-seven patients reported abdominal pain, 12 patients experienced bloody diarrhea, and 29 patients occult gastrointestinal bleeding. Six patients developed ileus, and in three patients, a bowel perforation occurred. Overall, 15/58 (25.9%) patients with GI involvement experienced severe GI involvement identified as severe bloody diarrhea, ileus, or bowel perforation.

Kidney involvement developed in 83 (38.8%) patients. Fourteen (6.5%) patients had macrohematuria, and the remaining, microhematuria. Proteinuria developed in 50 (23.4%) patients. Fifteen patients had daily proteinuria from 1.0 to 3.5 g. Nephrotic range proteinuria was detected in 13 patients. Acute kidney injury developed as a result of vasculitis in 23 (10.7%) patients. Overall, 30 (14.0%) patients had a severe course of kidney involvement, defined as the development of acute kidney injury or nephrotic syndrome. A renal biopsy was performed in 9 (4.2%) patients. Proliferative glomerulonephritis was noted in 5 patients, crescentic glomerulonephritis in 3 cases, and predominantly sclerotic glomerular lesions in one patient.

**Fig. 1:**
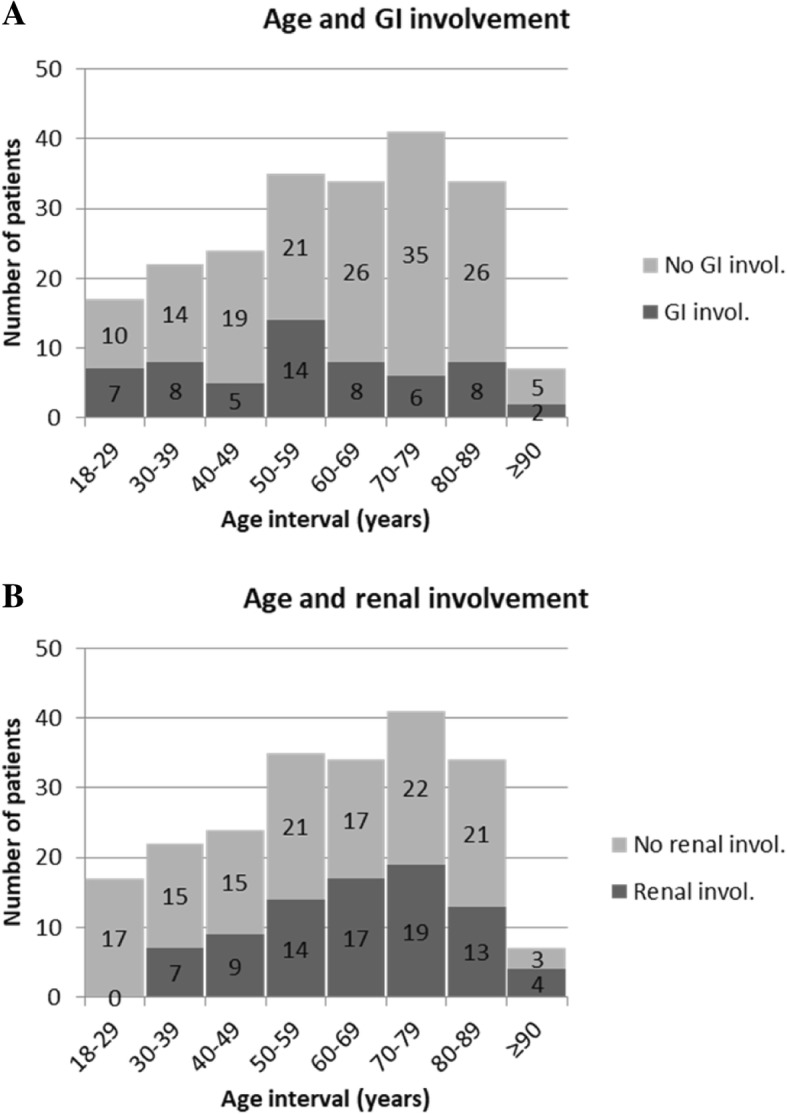
Age-related distribution of the adult IgAV patients with gastrointestinal (**a**) and renal (**b**) involvement

The characteristics of IgAV patients with and without GI or renal involvement are presented in Table 2. The subgroup of patients with renal involvement was significantly older compared to the subgroup with GI involvement (median (IQR) age 67.8 (53.6–77.4) vs. 55.5 (39.0–74.0) years, *p* = 0.007). The age-related distributions in patients with GI involvement and renal involvement are presented in Fig. 1.

**Table 2:** Characteristics of IgAV patients with and without gastrointestinal or renal involvement

Characteristics	Gastrointestinal involvement			Renal involvement		
	Yes	No	*p* value	Yes	No	*p* value
Number	58	156		83	131	
Disease duration (days)^#^	8 (7–15)	7 (5–21)	0.650	7 (5–19)	7 (5–21)	0.682
Age (years)^#^	55.5 (39.0–74.0)	65.4 (48.1–76.9)	0.053	67.8 (53.6–77.4)	59.5 (40.2–75.8)	0.006
Male gender*	70.7	55.1	0.043	61.4	58.0	0.669
Active smoking*	22.4	18.6	0.563	26.5	15.3	0.052
Prior infection*	34.5	31.4	0.743	32.5	32.1	1.0
Concurrent infection*	10.3	17.3	0.287	16.9	14.5	0.699
New medication*	25.9	23.7	0.724	24.1	24.4	1.0
Past cancer*	6.9	14.7	0.165	12.0	13.0	1.0
Generalized purpura*	79.3	40.4	< 0.001	60.2	45.0	0.036
Necrotic purpura*	46.6	45.5	1.0	48.2	44.3	0.673
Elevated serum IgA*	34.5	52.6	0.021	60.2	39.7	0.005
NLR^#^	4.89 (3.3–6.7)	3.8 (2.4–5.5)	0.003	4.6 (2.9–6.5)	3.8 (2.4–5.5)	0.041

^#^Median (IQR)

*Percentage

We measured the serum concentration of IgA in all patients. The pre-treatment IgA serum concentration exceeded the upper limit of reference in 102 (47.7%) cases. The median (IQR) concentration in patients with an elevated serum IgA level was 5.26 (4.60–6.70) g/l (normal range 0.61–3.56 g/l). No patient had a subnormal serum IgA concentration. IgAV patients with renal involvement had significantly higher serum IgA concentrations compared to the subgroup with a GI involvement (median (IQR) of 4.4 (3.2–5.6) vs. 3.6 (2.4–4.6) g/l, *p* = 0.008).

Median (IQR) pre-treatment NLR in our IgAV cohort was 4.0 (2.6–5.9), range 0.8–55.0. The ROC curve analysis demonstrated NLR > 3.5 as the best cut-off value for the prediction of concurrent GI or renal involvement with 68.7% sensitivity, 52.5% specificity, and the area under the curve of 0.606 (95%CI 0.537–0.672).

### IgAV treatment

We treated the patients in line with the local practice. Indications for systemic immunosuppressive treatment were necrotic purpura, any GI involvement, or severe kidney involvement. IgAV spontaneously remitted in 48 (22.4%) cases. Topical steroids were the only treatment in 21 (9.8%) patients. We treated 61 (28.5%) patients with intravenous methylprednisolone pulses (MP; 125–1000 mg qd, for three consecutive days). In 17 patients, MP pulses were the only treatment, while the remaining 44 patients continued with oral glucocorticoids. We treated a total of 129 (60.3%) patients with oral MP in a median (IQR) initial dose of 0.5 (0.4–0.7) kg/body weight. Thirty (14.0%) patients were additionally treated with immunomodulatory therapy: cyclophosphamide (22, 10.3%), intravenous immunoglobulins (9, 4.2%), mycophenolate mofetil (1, 0.5%), rituximab (1, 0.5%), dapsone (2, 0.9%), and colchicine (2, 0.9%). Three (1.4%) patients had a plasma exchange, and 5 (2.5%) patients additionally received montelukast. Three patients (1.4%) with ischemic bowel perforation needed surgical intervention. Another two patients (0.9%) underwent explorative laparoscopy. None of the patients required hemodialysis. Three (1.4%) patients died during the acute phase of the disease: two died due to severe GI involvement and one due to pneumocystis pneumonia.

In the multivariate logistic regression analysis, severe IgAV (severe GI or severe renal involvement) (OR 8.86 (95%CI 3.24–24.27), *p* < 0.001), renal involvement of any type (4.53 (95%CI 1.51–13.54), *p* = 0.007), generalized purpura (OR 3.30 (95%CI 1.1–10.35), *p* = 0.040), and female gender (OR 0.23 (95%CI 0.07–0.76), *p* = 0.017) were extracted as factors associated with the use of immunomodulatory therapy besides glucocorticoids.

### Predicting GI or renal involvement in IgAV

In the multivariate logistic regression analysis adjusted for concurrent infection, generalized purpura (OR 6.74 (95%CI 3.18–14.31), *p* < 0.001), pre-treatment NLR > 3.5 (OR 2.78 (95%CI 1.34–5.75), *p* = 0.006), and elevated serum IgA level (OR 0.40 (95%CI 0.20–0.79), *p* = 0.009) were extracted as factors associated with any GI involvement (Table 3). In a subanalysis focused on 15 cases with severe GI involvement generalized purpura (OR 6.79 (95%CI 1.38–33.53), *p* = 0.019), NLR > 3.5 (OR 11.28 (95%CI 2.06–61.76), *p* = 0.005), history of antecedent infection (OR 3.74 (95%CI 1.09–12.85), *p* = 0.036), and age (OR 0.94 (95%CI 0.91–0.98), *p* = 0.001) emerged as factors associated with severe GI complications.

**Table 3:** Predictors of gastrointestinal and renal involvement using multiple logistic regression

Characteristics	GI involvement		Renal involvement		GI or renal involvement	
	OR (95%CI)	*p* value	OR (95%CI)	*p* value	OR (95%CI)	*p* value
Age	–	–	1.02 (1.01–1.04)	0.023	–	–
Active smoking	–	–	3.23 (1.50–6.98)	0.003	2.51 (1.16–5.45)	0.020
Generalized purpura	6.74 (3.18–4.31)	< 0.001	2.00 (1.08–3.61)	0.027	3.80 (2.10–6.88)	< 0.001
Elevated serum IgA	0.40 (0.20–0.79)	0.009	2.25 (1.21–4.18)	0.010	–	–
NLR > 3.5	2.78 (1.34–5.75)	0.006	1.96 (1.02–3.77)	0.044	3.02 (1.63–5.60)	< 0.001

*NLR* neutrophil to lymphocyte ratio

Active smoking (OR 3.23 (95%CI 1.50–6.98), *p* = 0.003), generalized purpura (OR 1.98 (95%CI 1.08–3.61), *p* = 0.027), elevated serum IgA (OR 2.25 (95%CI 1.21–4.18), *p* = 0.010), NLR > 3.5 (OR 1.96 (95%CI 1.02–3.77), *p* = 0.044), and increasing patient age (1.02 (95%CI 1.01–1.04), *p* = 0.012) were extracted as factors associated with any renal involvement (Table 3). Active smoking (OR 4.42 (95%CI 1.69–11.58), *p* = 0.002), generalized purpura (OR 2.41 (95%CI 1.03–5.61), *p* = 0.041), and increasing age (OR 1.04 (95%CI 1.01–1.07), *p* = 0.007), but not serum IgA, emerged also as factors associated with acute kidney injury or nephrotic syndrome.

The composite outcome of either GI or renal involvement was associated with generalized purpura (OR 3.80 (95%CI 2.10–6.88), *p* < 0.001), active smoking (OR 2.51 (95%CI 1.16–5.45), *p* = 0.020), and NLR > 3.5 (OR 3.02 (95%CI 1.63–5.60), *p* < 0.001) (Table 3).

Disease duration prior to the diagnosis, patient gender, history of prior infection, use of new medication, history of cancer, and necrotic skin purpura at presentation were not significantly associated with GI or renal involvement in adult IgAV.

## Discussion

In the present study, we evaluated predictors of GI and renal involvement, the two most commonly affected visceral organs in IgAV [10], in a prospectively collected cohort of adult IgAV patients. Although IgAV is usually considered a childhood disease with GI involvement reported in 50–75%, and renal involvement in 40–50% of cases [11], the disease is not uncommon in adults [12]. In addition, the disease is frequently severe in adults, compared to commonly uneventful disease course in children [2].

In the majority of cases, GI and kidney inflammation followed the development of skin lesions. Particularly, renal involvement can be delayed for several weeks and can be missed [13] and can progress to chronic kidney disease [2, 3]. In addition, severe GI involvement with bowel ischemia that leads to tissue necrosis and perforation or major bleeding represents the major source of morbidity and mortality in the acute phase of IgAV [14]. The risk for this devastating complication is especially increased in the elderly and the polymorbid patients. Therefore, the identification of markers predicting the two most frequent visceral manifestations of IgAV would considerably ease the routine clinical work.

Our study shows that a generalized purpura (i.e., purpura above the waistline), and not skin necroses, predicts both renal and GI involvement in adult IgAV. As previously shown, generalized purpura in smokers also predicted the severity of kidney and GI involvement [15]. Our present findings are in contrast with those of Cao et al.*,* and Poterucha et al., who found a correlation between necrotic purpura and visceral involvement, and the absence of a correlation with the extent of skin lesions and visceral involvement, respectively [4, 5]. Nevertheless, similarly to Poterucha et al., we noticed a trend toward opposing age-related distribution between patients with GI and those with renal involvement—adult patients with GI involvement being significantly younger than those with renal involvement [5].

Active smoking emerged as a potential risk factor for renal involvement in adult IgAV. The association between smoking and the progression of chronic kidney disease has been appreciated in diabetic and several non-diabetic chronic kidney diseases, including IgA nephropathy [16, 17]. Cha et al. demonstrated a link between the progression of IgA nephropathy and smoking-related injury of the microvasculature [16]. In animal and in vitro models, nicotine activated inflammatory mediators, induced mesangial cell proliferation, and fibronectin production [18].

In our study, the serum IgA concentrations were divergent in patients with GI and renal involvement. Patients with the elevated serum IgA seem to have a lower risk of GI tract involvement but a higher risk of renal involvement. A lower frequency of elevated serum IgA in GI involvement has not been previously reported. Data in the literature is scarce; nevertheless, a recent study in children found significantly lower serum IgA in IgAV patients with scrotal involvement versus those without this manifestation [19]. And a study on biomarkers of childhood IgAV nephritis showed no significant differences in the serum IgA level comparing cases with and without nephritis [20].

The predictive roles of elevated pre-treatment NLR as a marker of systemic involvement in IgAV and a marker reflecting IgAV inflammatory response have been reported. Nagy et al. found that pre-treatment NLR > 3.3 correlated with the severity of the disease in patients who developed GI and renal involvement [7]. Similarly, we also found that higher NLR was associated with GI and renal involvement. The optimal cut-off in our cohort was NLR > 3.5. However, as it had poor sensitivity and specificity, it should not be interpreted in isolation.

Our study had several strengths. We prospectively investigated 214 adult patients with IgAV proven by skin biopsy and elucidated risk factors of severe involvement. However, the present study also had limitations: (1) cumulative smoking exposure, (2) a factor of passive smoking, and (3) long-term IgAV prognosis have not been analyzed due to lack of information. Because of the small number of patients who underwent renal biopsy, the histological features of kidney involvement could not be analyzed.

## Conclusion

Gastrointestinal or renal involvement in adult IgAV could be predicted by smoking status, distribution of skin lesions, and the neutrophil to lymphocyte ratio. The presented findings could help physicians identify the patients at risk who need vigilant monitoring during the acute phase of the disease.

## Data Availability

The datasets analyzed in the current study are available from the corresponding author on request.

## Abbreviations

CI: Confidence interval
EULAR/PRINTO/PRES: European League Against Rheumatism/Paediatric Rheumatology International Trials Organization/Paediatric Rheumatology European Society
GI: Gastrointestinal
IgAV: Immunoglobulin A vasculitis
IQR: Interquartile range
MP: Methylprednisolone
NLR: Neutrophil to lymphocyte ratio
OR: Odds ratio
ROC: Receiver operating characteristic
SD: Standard deviation
WBC: White blood cell count
